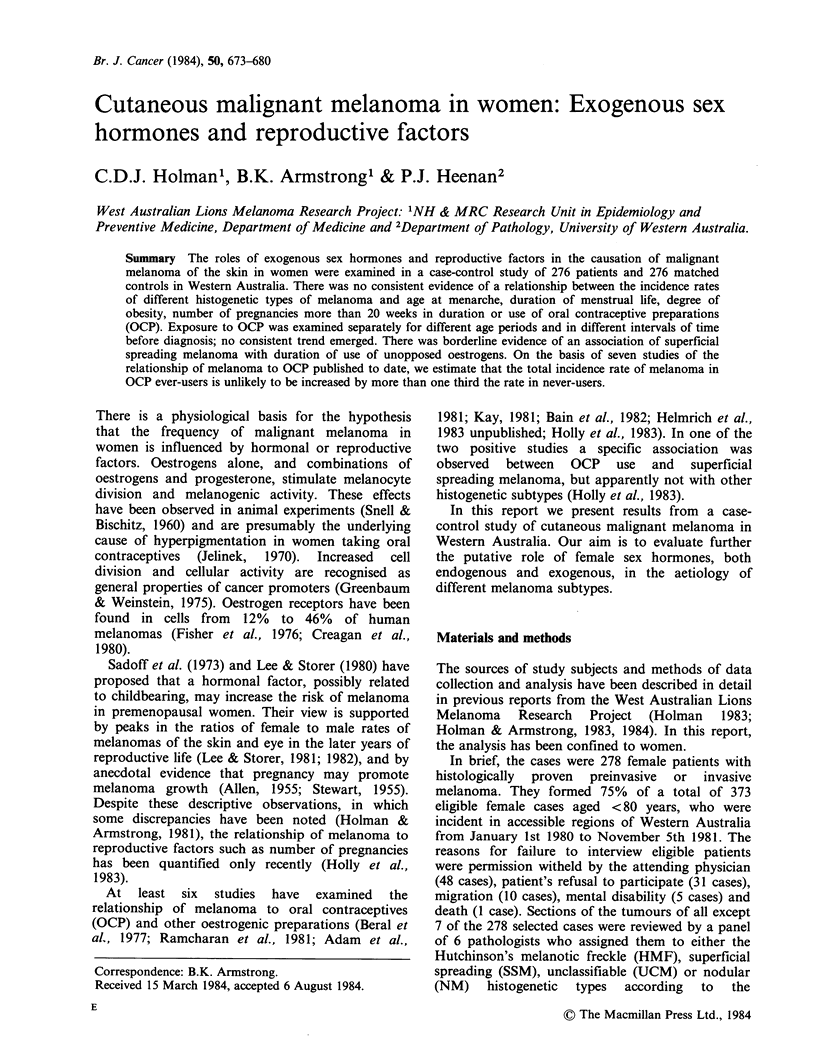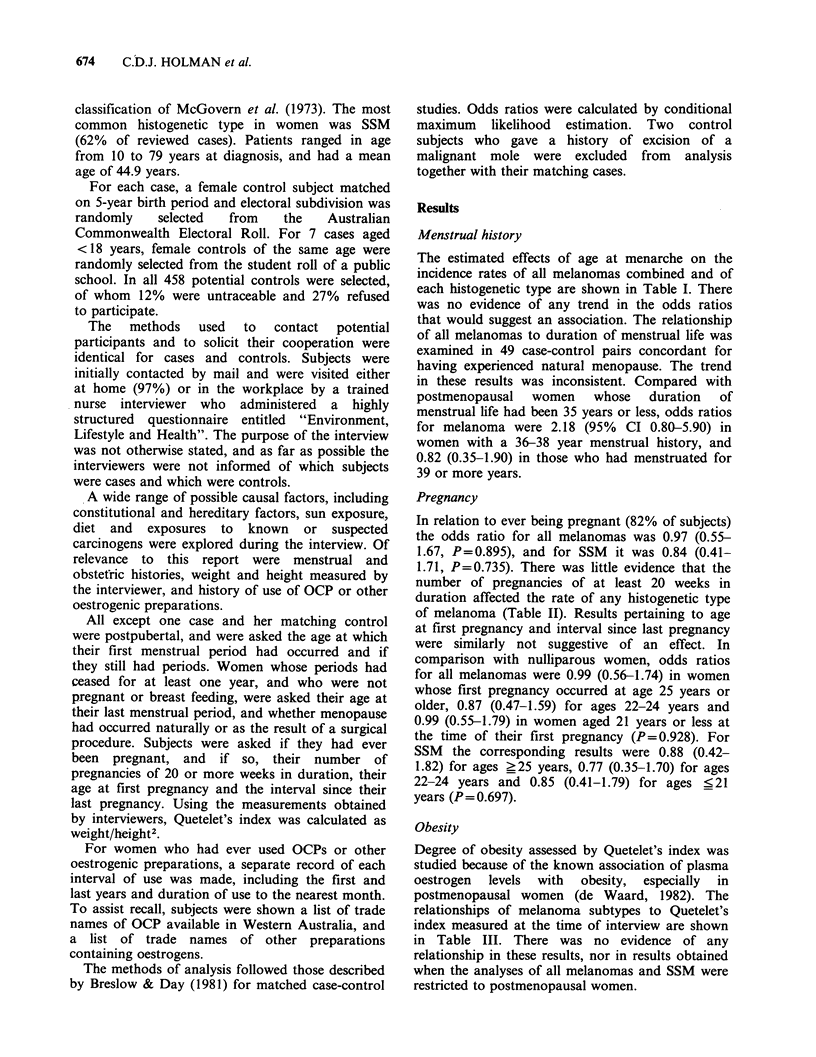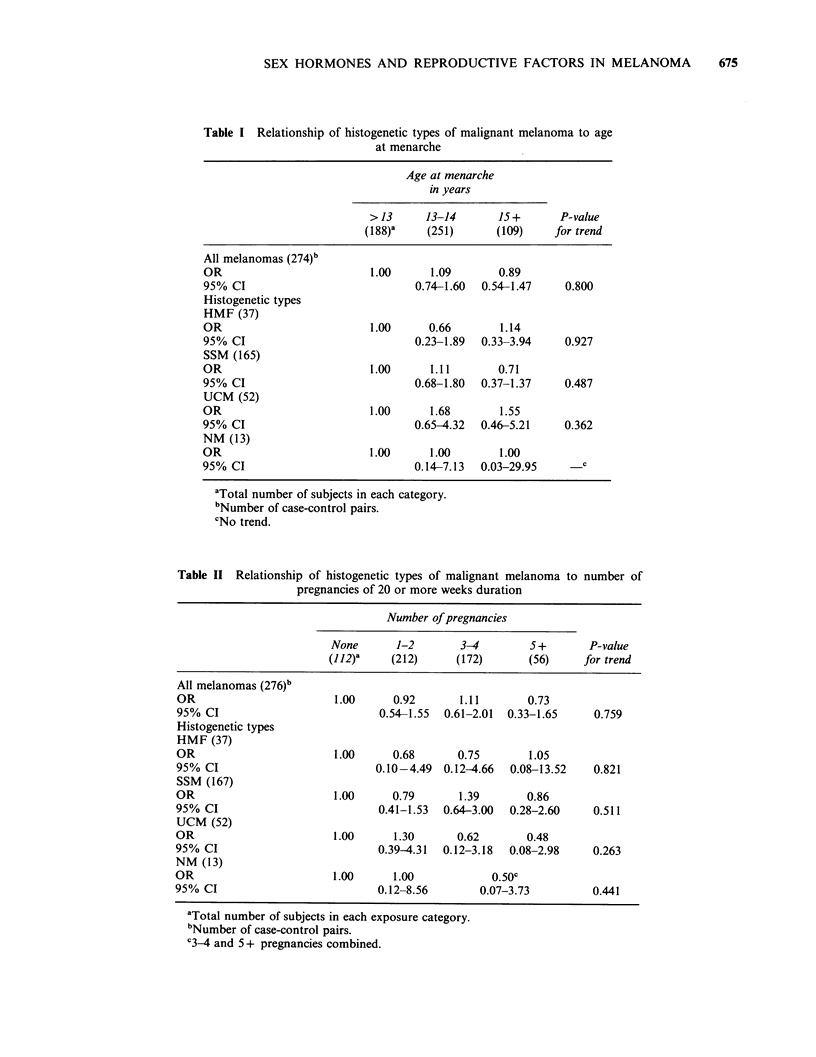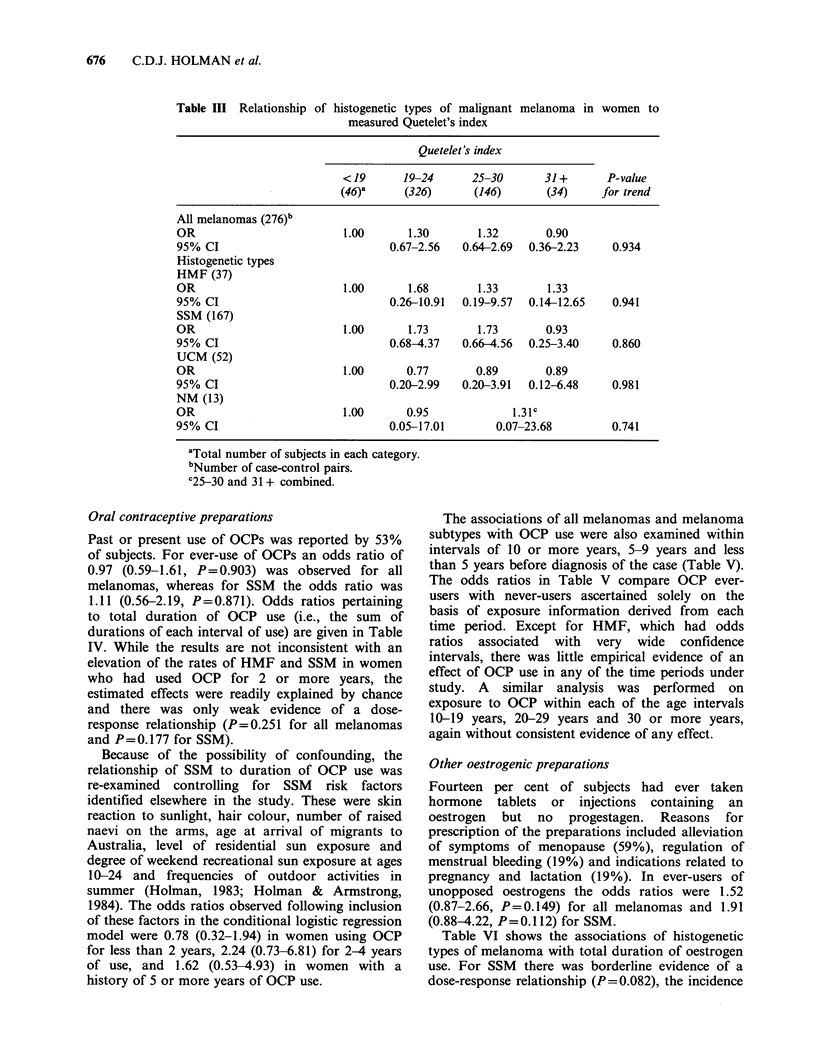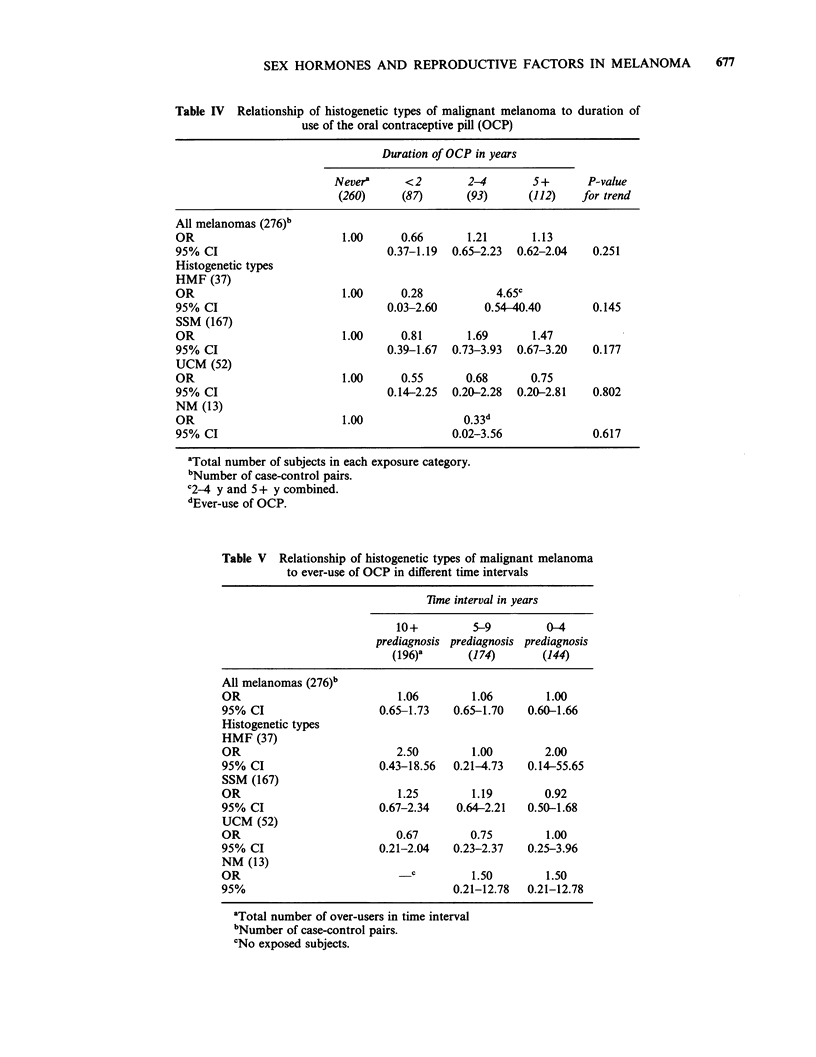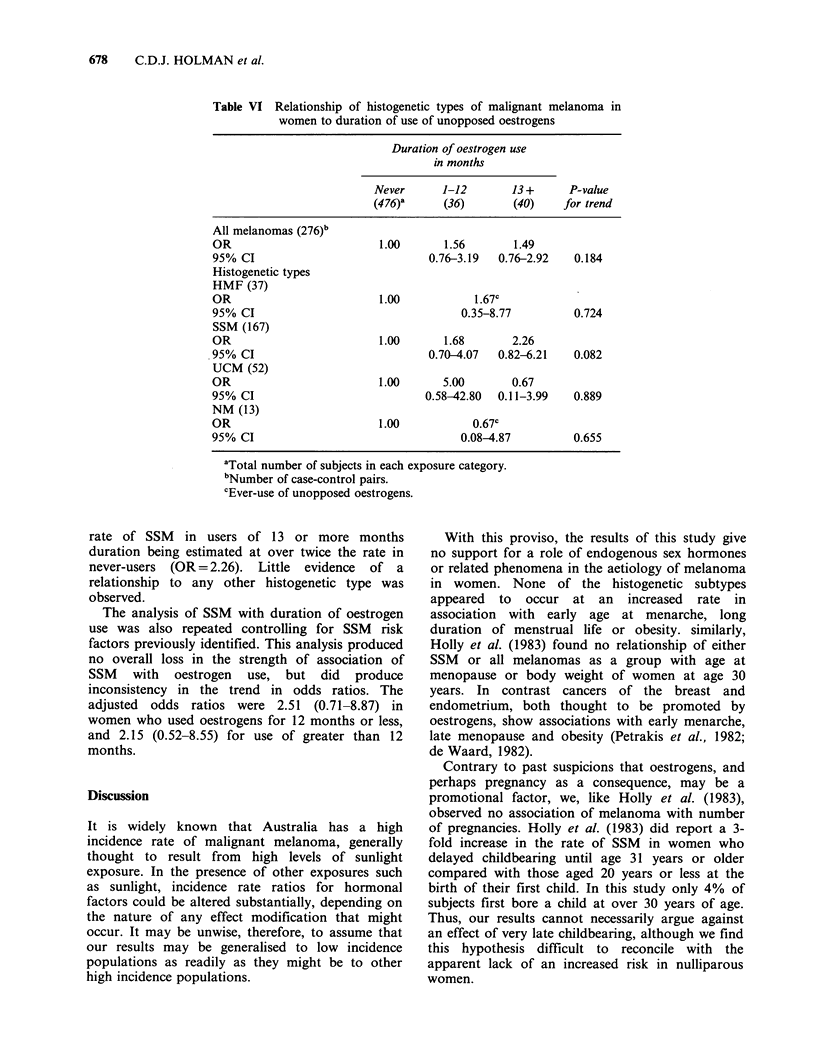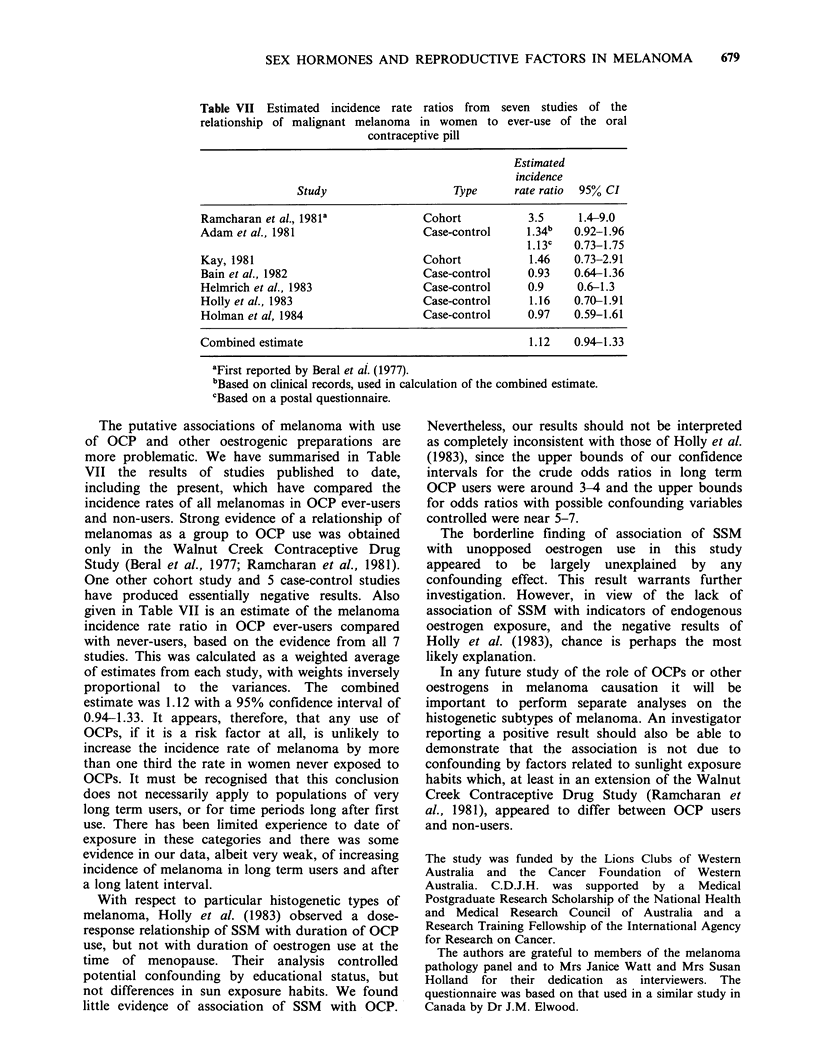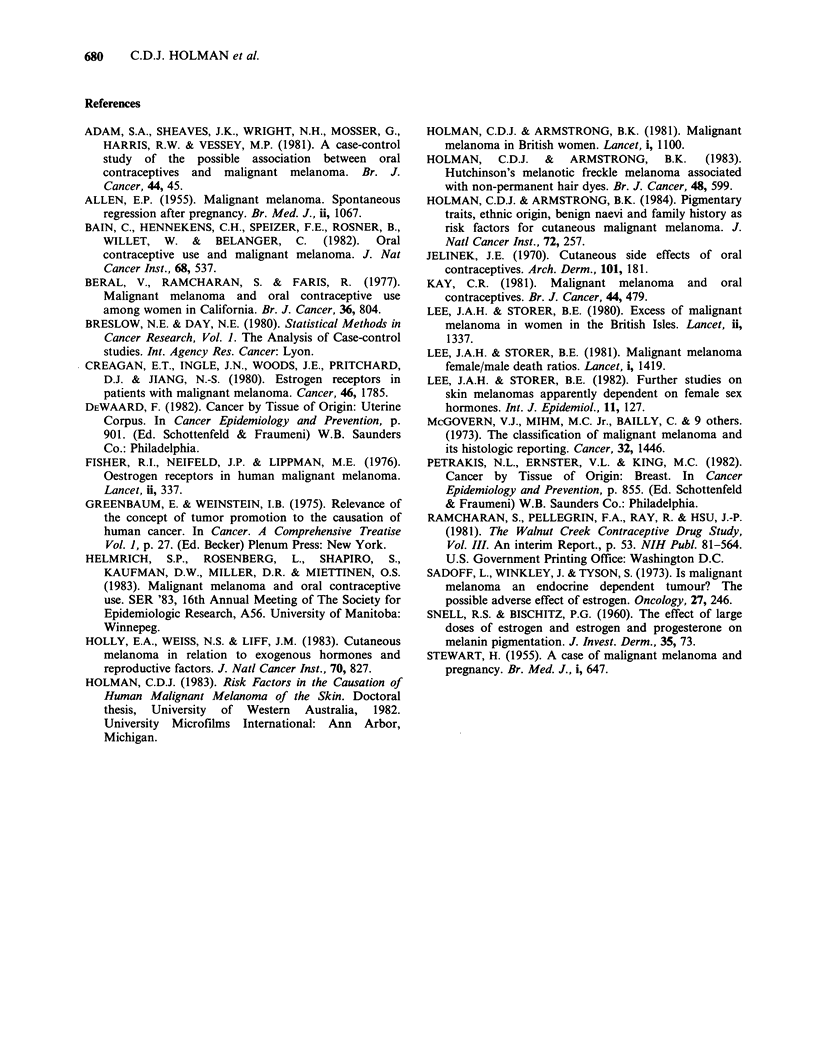# Cutaneous malignant melanoma in women: exogenous sex hormones and reproductive factors.

**DOI:** 10.1038/bjc.1984.235

**Published:** 1984-11

**Authors:** C. D. Holman, B. K. Armstrong, P. J. Heenan

## Abstract

The roles of exogenous sex hormones and reproductive factors in the causation of malignant melanoma of the skin in women were examined in a case-control study of 276 patients and 276 matched controls in Western Australia. There was no consistent evidence of a relationship between the incidence rates of different histogenetic types of melanoma and age at menarche, duration of menstrual life, degree of obesity, number of pregnancies more than 20 weeks in duration or use of oral contraceptive preparations (OCP). Exposure to OCP was examined separately for different age periods and in different intervals of time before diagnosis; no consistent trend emerged. There was borderline evidence of an association of superficial spreading melanoma with duration of use of unopposed oestrogens. On the basis of seven studies of the relationship of melanoma to OCP published to date, we estimate that the total incidence rate of melanoma in OCP ever-users is unlikely to be increased by more than one third the rate in never-users.


*Br. J. Cancer* (1984), **50**, 673–680

### Cutaneous malignant melanoma in women: Exogenous sex hormones and reproductive factors

C.D.J. Holman[1], B.K. Armstrong[1] & P.J. Heenan[2]

*West Australian Lions Melanoma Research Project: [1]NH & MRC Research Unit in Epidemiology and Preventive Medicine, Department of Medicine and [2]Department of Pathology, University of Western Australia.*

**Summary** The roles of exogenous sex hormones and reproductive factors in the causation of malignant melanoma of the skin in women were examined in a case-control study of 276 patients and 276 matched controls in Western Australia. There was no consistent evidence of a relationship between the incidence rates of different histogenetic types of melanoma and age at menarche, duration of menstrual life, degree of obesity, number of pregnancies more than 20 weeks in duration or use of oral contraceptive preparations (OCP). Exposure to OCP was examined separately for different age periods and in different intervals of time before diagnosis; no consistent trend emerged. There was borderline evidence of an association of superficial spreading melanoma with duration of use of unopposed oestrogens. On the basis of seven studies of the relationship of melanoma to OCP published to date, we estimate that the total incidence rate of melanoma in OCP ever-users is unlikely to be increased by more than one third the rate in never-users.

There is a physiological basis for the hypothesis that the frequency of malignant melanoma in women is influenced by hormonal or reproductive factors. Oestrogens alone, and combinations of oestrogens and progesterone, stimulate melanocyte division and melanogenic activity. These effects have been observed in animal experiments (Snell & Bischitz, 1960) and are presumably the underlying cause of hyperpigmentation in women taking oral contraceptives (Jelinek, 1970). Increased cell division and cellular activity are recognised as general properties of cancer promoters (Greenbaum & Weinstein, 1975). Oestrogen receptors have been found in cells from 12% to 46% of human melanomas (Fisher *et al.*, 1976; Creagan *et al.*, 1980).

Sadoff *et al.* (1973) and Lee & Storer (1980) have proposed that a hormonal factor, possibly related to childbearing, may increase the risk of melanoma in premenopausal women. Their view is supported by peaks in the ratios of female to male rates of melanomas of the skin and eye in the later years of reproductive life (Lee & Storer, 1981; 1982), and by anecdotal evidence that pregnancy may promote melanoma growth (Allen, 1955; Stewart, 1955). Despite these descriptive observations, in which some discrepancies have been noted (Holman & Armstrong, 1981), the relationship of melanoma to reproductive factors such as number of pregnancies has been quantified only recently (Holly *et al.*, 1983).

At least six studies have examined the relationship of melanoma to oral contraceptives (OCP) and other oestrogenic preparations (Beral *et al.*, 1977; Ramcharan *et al.*, 1981; Adam *et al.*, 1981; Kay, 1981; Bain *et al.*, 1982; Helmrich *et al.*, 1983 unpublished; Holly *et al.*, 1983). In one of the two positive studies a specific association was observed between OCP use and superficial spreading melanoma, but apparently not with other histogenetic subtypes (Holly *et al.*, 1983).

In this report we present results from a case-control study of cutaneous malignant melanoma in Western Australia. Our aim is to evaluate further the putative role of female sex hormones, both endogenous and exogenous, in the aetiology of different melanoma subtypes.

### Materials and methods

The sources of study subjects and methods of data collection and analysis have been described in detail in previous reports from the West Australian Lions Melanoma Research Project (Holman 1983; Holman & Armstrong, 1983, 1984). In this report, the analysis has been confined to women.

In brief, the cases were 278 female patients with histologically proven preinvasive or invasive melanoma. They formed 75% of a total of 373 eligible female cases aged <80 years, who were incident in accessible regions of Western Australia from January 1st 1980 to November 5th 1981. The reasons for failure to interview eligible patients were permission witheld by the attending physician (48 cases), patient's refusal to participate (31 cases), migration (10 cases), mental disability (5 cases) and death (1 case). Sections of the tumours of all except 7 of the 278 selected cases were reviewed by a panel of 6 pathologists who assigned them to either the Hutchinson's melanotic freckle (HMF), superficial spreading (SSM), unclassifiable (UCM) or nodular (NM) histogenetic types according to the

Correspondence: B.K. Armstrong.
Received 15 March 1984, accepted 6 August 1984.

© The Macmillan Press Ltd., 1984

classification of McGovern *et al.* (1973). The most common histogenetic type in women was SSM (62% of reviewed cases). Patients ranged in age from 10 to 79 years at diagnosis, and had a mean age of 44.9 years.

For each case, a female control subject matched on 5-year birth period and electoral subdivision was randomly selected from the Australian Commonwealth Electoral Roll. For 7 cases aged <18 years, female controls of the same age were randomly selected from the student roll of a public school. In all 458 potential controls were selected, of whom 12% were untraceable and 27% refused to participate.

The methods used to contact potential participants and to solicit their cooperation were identical for cases and controls. Subjects were initially contacted by mail and were visited either at home (97%) or in the workplace by a trained nurse interviewer who administered a highly structured questionnaire entitled "Environment, Lifestyle and Health". The purpose of the interview was not otherwise stated, and as far as possible the interviewers were not informed of which subjects were cases and which were controls.

A wide range of possible causal factors, including constitutional and hereditary factors, sun exposure, diet and exposures to known or suspected carcinogens were explored during the interview. Of relevance to this report were menstrual and obstetric histories, weight and height measured by the interviewer, and history of use of OCP or other oestrogenic preparations.

All except one case and her matching control were postpubertal, and were asked the age at which their first menstrual period had occurred and if they still had periods. Women whose periods had ceased for at least one year, and who were not pregnant or breast feeding, were asked their age at their last menstrual period, and whether menopause had occurred naturally or as the result of a surgical procedure. Subjects were asked if they had ever been pregnant, and if so, their number of pregnancies of 20 or more weeks in duration, their age at first pregnancy and the interval since their last pregnancy. Using the measurements obtained by interviewers, Quetelet's index was calculated as weight/height$^2$.

For women who had ever used OCPs or other oestrogenic preparations, a separate record of each interval of use was made, including the first and last years and duration of use to the nearest month. To assist recall, subjects were shown a list of trade names of OCP available in Western Australia, and a list of trade names of other preparations containing oestrogens.

The methods of analysis followed those described by Breslow & Day (1981) for matched case-control studies. Odds ratios were calculated by conditional maximum likelihood estimation. Two control subjects who gave a history of excision of a malignant mole were excluded from analysis together with their matching cases.

### Results

### *Menstrual history*

The estimated effects of age at menarche on the incidence rates of all melanomas combined and of each histogenetic type are shown in Table I. There was no evidence of any trend in the odds ratios that would suggest an association. The relationship of all melanomas to duration of menstrual life was examined in 49 case-control pairs concordant for having experienced natural menopause. The trend in these results was inconsistent. Compared with postmenopausal women whose duration of menstrual life had been 35 years or less, odds ratios for melanoma were 2.18 (95% CI 0.80–5.90) in women with a 36–38 year menstrual history, and 0.82 (0.35–1.90) in those who had menstruated for 39 or more years.

### *Pregnancy*

In relation to ever being pregnant (82% of subjects) the odds ratio for all melanomas was 0.97 (0.55–1.67, $P=0.895$), and for SSM it was 0.84 (0.41–1.71, $P=0.735$). There was little evidence that the number of pregnancies of at least 20 weeks in duration affected the rate of any histogenetic type of melanoma (Table II). Results pertaining to age at first pregnancy and interval since last pregnancy were similarly not suggestive of an effect. In comparison with nulliparous women, odds ratios for all melanomas were 0.99 (0.56–1.74) in women whose first pregnancy occurred at age 25 years or older, 0.87 (0.47–1.59) for ages 22–24 years and 0.99 (0.55–1.79) in women aged 21 years or less at the time of their first pregnancy ($P=0.928$). For SSM the corresponding results were 0.88 (0.42–1.82) for ages $\geqq 25$ years, 0.77 (0.35–1.70) for ages 22–24 years and 0.85 (0.41–1.79) for ages $\leqq 21$ years ($P=0.697$).

### *Obesity*

Degree of obesity assessed by Quetelet's index was studied because of the known association of plasma oestrogen levels with obesity, especially in postmenopausal women (de Waard, 1982). The relationships of melanoma subtypes to Quetelet's index measured at the time of interview are shown in Table III. There was no evidence of any relationship in these results, nor in results obtained when the analyses of all melanomas and SSM were restricted to postmenopausal women.

**Table I** Relationship of histogenetic types of malignant melanoma to age at menarche

| | *Age at menarche in years* | | | |
|---|---|---|---|---|
| | *>13* (188)[a] | *13–14* (251) | *15+* (109) | *P-value for trend* |
| All melanomas (274)[b] | | | | |
| OR | 1.00 | 1.09 | 0.89 | |
| 95% CI | | 0.74–1.60 | 0.54–1.47 | 0.800 |
| Histogenetic types | | | | |
| HMF (37) | | | | |
| OR | 1.00 | 0.66 | 1.14 | |
| 95% CI | | 0.23–1.89 | 0.33–3.94 | 0.927 |
| SSM (165) | | | | |
| OR | 1.00 | 1.11 | 0.71 | |
| 95% CI | | 0.68–1.80 | 0.37–1.37 | 0.487 |
| UCM (52) | | | | |
| OR | 1.00 | 1.68 | 1.55 | |
| 95% CI | | 0.65–4.32 | 0.46–5.21 | 0.362 |
| NM (13) | | | | |
| OR | 1.00 | 1.00 | 1.00 | |
| 95% CI | | 0.14–7.13 | 0.03–29.95 | —[c] |

[a]Total number of subjects in each category.
[b]Number of case-control pairs.
[c]No trend.

**Table II** Relationship of histogenetic types of malignant melanoma to number of pregnancies of 20 or more weeks duration

| | *Number of pregnancies* | | | | |
|---|---|---|---|---|---|
| | *None* (*112*)[a] | *1–2* (212) | *3–4* (172) | *5+* (56) | *P-value for trend* |
| All melanomas (276)[b] | | | | | |
| OR | 1.00 | 0.92 | 1.11 | 0.73 | |
| 95% CI | | 0.54–1.55 | 0.61–2.01 | 0.33–1.65 | 0.759 |
| Histogenetic types | | | | | |
| HMF (37) | | | | | |
| OR | 1.00 | 0.68 | 0.75 | 1.05 | |
| 95% CI | | 0.10–4.49 | 0.12–4.66 | 0.08–13.52 | 0.821 |
| SSM (167) | | | | | |
| OR | 1.00 | 0.79 | 1.39 | 0.86 | |
| 95% CI | | 0.41–1.53 | 0.64–3.00 | 0.28–2.60 | 0.511 |
| UCM (52) | | | | | |
| OR | 1.00 | 1.30 | 0.62 | 0.48 | |
| 95% CI | | 0.39–4.31 | 0.12–3.18 | 0.08–2.98 | 0.263 |
| NM (13) | | | | | |
| OR | 1.00 | 1.00 | 0.50[c] | | |
| 95% CI | | 0.12–8.56 | 0.07–3.73 | | 0.441 |

[a]Total number of subjects in each exposure category.
[b]Number of case-control pairs.
[c]3–4 and 5+ pregnancies combined.

**Table III** Relationship of histogenetic types of malignant melanoma in women to measured Quetelet's index

| | *Quetelet's index* | | | | |
|---|---|---|---|---|---|
| | *< 19 (46)*[a] | *19–24 (326)* | *25–30 (146)* | *31 + (34)* | *P-value for trend* |
| All melanomas (276)[b] | | | | | |
| OR | 1.00 | 1.30 | 1.32 | 0.90 | |
| 95% CI | | 0.67–2.56 | 0.64–2.69 | 0.36–2.23 | 0.934 |
| Histogenetic types | | | | | |
| HMF (37) | | | | | |
| OR | 1.00 | 1.68 | 1.33 | 1.33 | |
| 95% CI | | 0.26–10.91 | 0.19–9.57 | 0.14–12.65 | 0.941 |
| SSM (167) | | | | | |
| OR | 1.00 | 1.73 | 1.73 | 0.93 | |
| 95% CI | | 0.68–4.37 | 0.66–4.56 | 0.25–3.40 | 0.860 |
| UCM (52) | | | | | |
| OR | 1.00 | 0.77 | 0.89 | 0.89 | |
| 95% CI | | 0.20–2.99 | 0.20–3.91 | 0.12–6.48 | 0.981 |
| NM (13) | | | | | |
| OR | 1.00 | 0.95 | 1.31[c] | | |
| 95% CI | | 0.05–17.01 | 0.07–23.68 | | 0.741 |

[a]Total number of subjects in each category.
[b]Number of case-control pairs.
[c]25–30 and 31 + combined.

*Oral contraceptive preparations*

Past or present use of OCPs was reported by 53% of subjects. For ever-use of OCPs an odds ratio of 0.97 (0.59–1.61, $P = 0.903$) was observed for all melanomas, whereas for SSM the odds ratio was 1.11 (0.56–2.19, $P = 0.871$). Odds ratios pertaining to total duration of OCP use (i.e., the sum of durations of each interval of use) are given in Table IV. While the results are not inconsistent with an elevation of the rates of HMF and SSM in women who had used OCP for 2 or more years, the estimated effects were readily explained by chance and there was only weak evidence of a dose-response relationship ($P = 0.251$ for all melanomas and $P = 0.177$ for SSM).

Because of the possibility of confounding, the relationship of SSM to duration of OCP use was re-examined controlling for SSM risk factors identified elsewhere in the study. These were skin reaction to sunlight, hair colour, number of raised naevi on the arms, age at arrival of migrants to Australia, level of residential sun exposure and degree of weekend recreational sun exposure at ages 10–24 and frequencies of outdoor activities in summer (Holman, 1983; Holman & Armstrong, 1984). The odds ratios observed following inclusion of these factors in the conditional logistic regression model were 0.78 (0.32–1.94) in women using OCP for less than 2 years, 2.24 (0.73–6.81) for 2–4 years of use, and 1.62 (0.53–4.93) in women with a history of 5 or more years of OCP use.

The associations of all melanomas and melanoma subtypes with OCP use were also examined within intervals of 10 or more years, 5–9 years and less than 5 years before diagnosis of the case (Table V). The odds ratios in Table V compare OCP ever-users with never-users ascertained solely on the basis of exposure information derived from each time period. Except for HMF, which had odds ratios associated with very wide confidence intervals, there was little empirical evidence of an effect of OCP use in any of the time periods under study. A similar analysis was performed on exposure to OCP within each of the age intervals 10–19 years, 20–29 years and 30 or more years, again without consistent evidence of any effect.

*Other oestrogenic preparations*

Fourteen per cent of subjects had ever taken hormone tablets or injections containing an oestrogen but no progestagen. Reasons for prescription of the preparations included alleviation of symptoms of menopause (59%), regulation of menstrual bleeding (19%) and indications related to pregnancy and lactation (19%). In ever-users of unopposed oestrogens the odds ratios were 1.52 (0.87–2.66, $P = 0.149$) for all melanomas and 1.91 (0.88–4.22, $P = 0.112$) for SSM.

Table VI shows the associations of histogenetic types of melanoma with total duration of oestrogen use. For SSM there was borderline evidence of a dose-response relationship ($P = 0.082$), the incidence

**Table IV** Relationship of histogenetic types of malignant melanoma to duration of use of the oral contraceptive pill (OCP)

| | Duration of OCP in years | | | | |
|---|---|---|---|---|---|
| | *Never*[a] *(260)* | *<2 (87)* | *2–4 (93)* | *5+ (112)* | *P-value for trend* |
| All melanomas (276)[b] | | | | | |
| OR | 1.00 | 0.66 | 1.21 | 1.13 | |
| 95% CI | | 0.37–1.19 | 0.65–2.23 | 0.62–2.04 | 0.251 |
| Histogenetic types | | | | | |
| HMF (37) | | | | | |
| OR | 1.00 | 0.28 | 4.65[c] | | |
| 95% CI | | 0.03–2.60 | 0.54–40.40 | | 0.145 |
| SSM (167) | | | | | |
| OR | 1.00 | 0.81 | 1.69 | 1.47 | |
| 95% CI | | 0.39–1.67 | 0.73–3.93 | 0.67–3.20 | 0.177 |
| UCM (52) | | | | | |
| OR | 1.00 | 0.55 | 0.68 | 0.75 | |
| 95% CI | | 0.14–2.25 | 0.20–2.28 | 0.20–2.81 | 0.802 |
| NM (13) | | | | | |
| OR | 1.00 | | 0.33[d] | | |
| 95% CI | | | 0.02–3.56 | | 0.617 |

[a]Total number of subjects in each exposure category.
[b]Number of case-control pairs.
[c]2–4 y and 5+ y combined.
[d]Ever-use of OCP.

**Table V** Relationship of histogenetic types of malignant melanoma to ever-use of OCP in different time intervals

| | Time interval in years | | |
|---|---|---|---|
| | 10+ *prediagnosis* (196)[a] | 5–9 *prediagnosis (174)* | 0–4 *prediagnosis (144)* |
| All melanomas (276)[b] | | | |
| OR | 1.06 | 1.06 | 1.00 |
| 95% CI | 0.65–1.73 | 0.65–1.70 | 0.60–1.66 |
| Histogenetic types | | | |
| HMF (37) | | | |
| OR | 2.50 | 1.00 | 2.00 |
| 95% CI | 0.43–18.56 | 0.21–4.73 | 0.14–55.65 |
| SSM (167) | | | |
| OR | 1.25 | 1.19 | 0.92 |
| 95% CI | 0.67–2.34 | 0.64–2.21 | 0.50–1.68 |
| UCM (52) | | | |
| OR | 0.67 | 0.75 | 1.00 |
| 95% CI | 0.21–2.04 | 0.23–2.37 | 0.25–3.96 |
| NM (13) | | | |
| OR | —[c] | 1.50 | 1.50 |
| 95% | | 0.21–12.78 | 0.21–12.78 |

[a]Total number of over-users in time interval
[b]Number of case-control pairs.
[c]No exposed subjects.

**Table VI** Relationship of histogenetic types of malignant melanoma in women to duration of use of unopposed oestrogens

| | *Duration of oestrogen use in months* | | | |
|---|---|---|---|---|
| | *Never (476)*[a] | *1–12 (36)* | *13+ (40)* | *P-value for trend* |
| All melanomas (276)[b] | | | | |
| OR | 1.00 | 1.56 | 1.49 | |
| 95% CI | | 0.76–3.19 | 0.76–2.92 | 0.184 |
| Histogenetic types | | | | |
| HMF (37) | | | | |
| OR | 1.00 | 1.67[c] | | |
| 95% CI | | 0.35–8.77 | | 0.724 |
| SSM (167) | | | | |
| OR | 1.00 | 1.68 | 2.26 | |
| 95% CI | | 0.70–4.07 | 0.82–6.21 | 0.082 |
| UCM (52) | | | | |
| OR | 1.00 | 5.00 | 0.67 | |
| 95% CI | | 0.58–42.80 | 0.11–3.99 | 0.889 |
| NM (13) | | | | |
| OR | 1.00 | 0.67[c] | | |
| 95% CI | | 0.08–4.87 | | 0.655 |

[a]Total number of subjects in each exposure category.
[b]Number of case-control pairs.
[c]Ever-use of unopposed oestrogens.

rate of SSM in users of 13 or more months duration being estimated at over twice the rate in never-users (OR = 2.26). Little evidence of a relationship to any other histogenetic type was observed.

The analysis of SSM with duration of oestrogen use was also repeated controlling for SSM risk factors previously identified. This analysis produced no overall loss in the strength of association of SSM with oestrogen use, but did produce inconsistency in the trend in odds ratios. The adjusted odds ratios were 2.51 (0.71–8.87) in women who used oestrogens for 12 months or less, and 2.15 (0.52–8.55) for use of greater than 12 months.

### Discussion

It is widely known that Australia has a high incidence rate of malignant melanoma, generally thought to result from high levels of sunlight exposure. In the presence of other exposures such as sunlight, incidence rate ratios for hormonal factors could be altered substantially, depending on the nature of any effect modification that might occur. It may be unwise, therefore, to assume that our results may be generalised to low incidence populations as readily as they might be to other high incidence populations.

With this proviso, the results of this study give no support for a role of endogenous sex hormones or related phenomena in the aetiology of melanoma in women. None of the histogenetic subtypes appeared to occur at an increased rate in association with early age at menarche, long duration of menstrual life or obesity. similarly, Holly *et al.* (1983) found no relationship of either SSM or all melanomas as a group with age at menopause or body weight of women at age 30 years. In contrast cancers of the breast and endometrium, both thought to be promoted by oestrogens, show associations with early menarche, late menopause and obesity (Petrakis *et al.*, 1982; de Waard, 1982).

Contrary to past suspicions that oestrogens, and perhaps pregnancy as a consequence, may be a promotional factor, we, like Holly *et al.* (1983), observed no association of melanoma with number of pregnancies. Holly *et al.* (1983) did report a 3-fold increase in the rate of SSM in women who delayed childbearing until age 31 years or older compared with those aged 20 years or less at the birth of their first child. In this study only 4% of subjects first bore a child at over 30 years of age. Thus, our results cannot necessarily argue against an effect of very late childbearing, although we find this hypothesis difficult to reconcile with the apparent lack of an increased risk in nulliparous women.

**Table VII** Estimated incidence rate ratios from seven studies of the relationship of malignant melanoma in women to ever-use of the oral contraceptive pill

| *Study* | *Type* | *Estimated incidence rate ratio* | *95% CI* |
|---|---|---|---|
| Ramcharan *et al.*, 1981[a] | Cohort | 3.5 | 1.4–9.0 |
| Adam *et al.*, 1981 | Case-control | 1.34[b] | 0.92–1.96 |
| | | 1.13[c] | 0.73–1.75 |
| Kay, 1981 | Cohort | 1.46 | 0.73–2.91 |
| Bain *et al.*, 1982 | Case-control | 0.93 | 0.64–1.36 |
| Helmrich *et al.*, 1983 | Case-control | 0.9 | 0.6–1.3 |
| Holly *et al.*, 1983 | Case-control | 1.16 | 0.70–1.91 |
| Holman *et al*, 1984 | Case-control | 0.97 | 0.59–1.61 |
| Combined estimate | | 1.12 | 0.94–1.33 |

[a]First reported by Beral *et al.* (1977).
[b]Based on clinical records, used in calculation of the combined estimate.
[c]Based on a postal questionnaire.

The putative associations of melanoma with use of OCP and other oestrogenic preparations are more problematic. We have summarised in Table VII the results of studies published to date, including the present, which have compared the incidence rates of all melanomas in OCP ever-users and non-users. Strong evidence of a relationship of melanomas as a group to OCP use was obtained only in the Walnut Creek Contraceptive Drug Study (Beral *et al.*, 1977; Ramcharan *et al.*, 1981). One other cohort study and 5 case-control studies have produced essentially negative results. Also given in Table VII is an estimate of the melanoma incidence rate ratio in OCP ever-users compared with never-users, based on the evidence from all 7 studies. This was calculated as a weighted average of estimates from each study, with weights inversely proportional to the variances. The combined estimate was 1.12 with a 95% confidence interval of 0.94–1.33. It appears, therefore, that any use of OCPs, if it is a risk factor at all, is unlikely to increase the incidence rate of melanoma by more than one third the rate in women never exposed to OCPs. It must be recognised that this conclusion does not necessarily apply to populations of very long term users, or for time periods long after first use. There has been limited experience to date of exposure in these categories and there was some evidence in our data, albeit very weak, of increasing incidence of melanoma in long term users and after a long latent interval.

With respect to particular histogenetic types of melanoma, Holly *et al.* (1983) observed a dose-response relationship of SSM with duration of OCP use, but not with duration of oestrogen use at the time of menopause. Their analysis controlled potential confounding by educational status, but not differences in sun exposure habits. We found little evidence of association of SSM with OCP. Nevertheless, our results should not be interpreted as completely inconsistent with those of Holly *et al.* (1983), since the upper bounds of our confidence intervals for the crude odds ratios in long term OCP users were around 3–4 and the upper bounds for odds ratios with possible confounding variables controlled were near 5–7.

The borderline finding of association of SSM with unopposed oestrogen use in this study appeared to be largely unexplained by any confounding effect. This result warrants further investigation. However, in view of the lack of association of SSM with indicators of endogenous oestrogen exposure, and the negative results of Holly *et al.* (1983), chance is perhaps the most likely explanation.

In any future study of the role of OCPs or other oestrogens in melanoma causation it will be important to perform separate analyses on the histogenetic subtypes of melanoma. An investigator reporting a positive result should also be able to demonstrate that the association is not due to confounding by factors related to sunlight exposure habits which, at least in an extension of the Walnut Creek Contraceptive Drug Study (Ramcharan *et al.*, 1981), appeared to differ between OCP users and non-users.

The study was funded by the Lions Clubs of Western Australia and the Cancer Foundation of Western Australia. C.D.J.H. was supported by a Medical Postgraduate Research Scholarship of the National Health and Medical Research Council of Australia and a Research Training Fellowship of the International Agency for Research on Cancer.

The authors are grateful to members of the melanoma pathology panel and to Mrs Janice Watt and Mrs Susan Holland for their dedication as interviewers. The questionnaire was based on that used in a similar study in Canada by Dr J.M. Elwood.

### References

ADAM, S.A., SHEAVES, J.K., WRIGHT, N.H., MOSSER, G., HARRIS, R.W. & VESSEY, M.P. (1981). A case-control study of the possible association between oral contraceptives and malignant melanoma. *Br. J. Cancer*, **44**, 45.

ALLEN, E.P. (1955). Malignant melanoma. Spontaneous regression after pregnancy. *Br. Med. J.*, **ii**, 1067.

BAIN, C., HENNEKENS, C.H., SPEIZER, F.E., ROSNER, B., WILLET, W. & BELANGER, C. (1982). Oral contraceptive use and malignant melanoma. *J. Nat Cancer Inst.*, **68**, 537.

BERAL, V., RAMCHARAN, S. & FARIS, R. (1977). Malignant melanoma and oral contraceptive use among women in California. *Br. J. Cancer*, **36**, 804.

BRESLOW, N.E. & DAY, N.E. (1980). *Statistical Methods in Cancer Research, Vol. 1.* The Analysis of Case-control studies. *Int. Agency Res. Cancer*: Lyon.

CREAGAN, E.T., INGLE, J.N., WOODS, J.E., PRITCHARD, D.J. & JIANG, N.-S. (1980). Estrogen receptors in patients with malignant melanoma. *Cancer*, **46**, 1785.

DEWAARD, F. (1982). Cancer by Tissue of Origin: Uterine Corpus. In *Cancer Epidemiology and Prevention*, p. 901. (Ed. Schottenfeld & Fraumeni) W.B. Saunders Co.: Philadelphia.

FISHER, R.I., NEIFELD, J.P. & LIPPMAN, M.E. (1976). Oestrogen receptors in human malignant melanoma. *Lancet*, **ii**, 337.

GREENBAUM, E. & WEINSTEIN, I.B. (1975). Relevance of the concept of tumor promotion to the causation of human cancer. In *Cancer. A Comprehensive Treatise Vol. 1*, p. 27. (Ed. Becker) Plenum Press: New York.

HELMRICH, S.P., ROSENBERG, L., SHAPIRO, S., KAUFMAN, D.W., MILLER, D.R. & MIETTINEN, O.S. (1983). Malignant melanoma and oral contraceptive use. SER '83, 16th Annual Meeting of The Society for Epidemiologic Research, A56. University of Manitoba: Winnepeg.

HOLLY, E.A., WEISS, N.S. & LIFF, J.M. (1983). Cutaneous melanoma in relation to exogenous hormones and reproductive factors. *J. Natl Cancer Inst.*, **70**, 827.

HOLMAN, C.D.J. (1983). *Risk Factors in the Causation of Human Malignant Melanoma of the Skin.* Doctoral thesis, University of Western Australia, 1982. University Microfilms International: Ann Arbor, Michigan.

HOLMAN, C.D.J. & ARMSTRONG, B.K. (1981). Malignant melanoma in British women. *Lancet*, **i**, 1100.

HOLMAN, C.D.J. & ARMSTRONG, B.K. (1983). Hutchinson's melanotic freckle melanoma associated with non-permanent hair dyes. *Br. J. Cancer*, **48**, 599.

HOLMAN, C.D.J. & ARMSTRONG, B.K. (1984). Pigmentary traits, ethnic origin, benign naevi and family history as risk factors for cutaneous malignant melanoma. *J. Natl Cancer Inst.*, **72**, 257.

JELINEK, J.E. (1970). Cutaneous side effects of oral contraceptives. *Arch. Derm.*, **101**, 181.

KAY, C.R. (1981). Malignant melanoma and oral contraceptives. *Br. J. Cancer*, **44**, 479.

LEE, J.A.H. & STORER, B.E. (1980). Excess of malignant melanoma in women in the British Isles. *Lancet*, **ii**, 1337.

LEE, J.A.H. & STORER, B.E. (1981). Malignant melanoma female/male death ratios. *Lancet*, **i**, 1419.

LEE, J.A.H. & STORER, B.E. (1982). Further studies on skin melanomas apparently dependent on female sex hormones. *Int. J. Epidemiol.*, **11**, 127.

McGOVERN, V.J., MIHM, M.C. Jr., BAILLY, C. & 9 others. (1973). The classification of malignant melanoma and its histologic reporting. *Cancer*, **32**, 1446.

PETRAKIS, N.L., ERNSTER, V.L. & KING, M.C. (1982). Cancer by Tissue of Origin: Breast. In *Cancer Epidemiology and Prevention*, p. 855. (Ed. Schottenfeld & Fraumeni) W.B. Saunders Co.: Philadelphia.

RAMCHARAN, S., PELLEGRIN, F.A., RAY, R. & HSU, J.-P. (1981). *The Walnut Creek Contraceptive Drug Study, Vol. III.* An interim Report., p. 53. *NIH Publ.* 81-564. U.S. Government Printing Office: Washington D.C.

SADOFF, L., WINKLEY, J. & TYSON, S. (1973). Is malignant melanoma an endocrine dependent tumour? The possible adverse effect of estrogen. *Oncology*, **27**, 246.

SNELL, R.S. & BISCHITZ, P.G. (1960). The effect of large doses of estrogen and estrogen and progesterone on melanin pigmentation. *J. Invest. Derm.*, **35**, 73.

STEWART, H. (1955). A case of malignant melanoma and pregnancy. *Br. Med. J.*, **i**, 647.